# Isobolographic analysis of the opioid-opioid interactions in a tonic and a phasic mouse model of induced nociceptive pain

**DOI:** 10.1186/s12929-014-0062-6

**Published:** 2014-07-15

**Authors:** Hugo F Miranda, Viviana Noriega, Pilar Zanetta, Juan Carlos Prieto, Juan Carlos Prieto-Rayo, Nicolás Aranda, Fernando Sierralta

**Affiliations:** 1 Pharmacology Program, ICBM, Faculty of Medicine, Universidad de Chile, Independencia 1027, Santiago, Chile; 2 Faculty of Medicine, School of Pharmacy, Universidad Andrés Bello, Av. República 252, Santiago, Chile; 3 Cardiovascular Department, Hospital Clínico, Universidad de Chile, Santos Dumont 999, Santiago, Chile; 4 Faculty of Odontology, Universidad Finis Terrae, Providencia 1509, Santiago, Chile

**Keywords:** Interaction opioid-opioid, Acetic acid writhing test, Hot plate assay, Isobolographic analysis, Synergism

## Abstract

**Background:**

Opioids have been used for the management of pain and coadministration of two opioids may induce synergism. In a model of tonic pain, the acetic acid writhing test and in a phasic model, the hot plate, the antinociceptive interaction between fentanyl, methadone, morphine, and tramadol was evaluated.

**Results:**

The potency of opioids in the writhing test compared to the hot plate assay was from 2.5 (fentanyl) to 15.5 (morphine) times, respectively. The ED_50_ was used in a fixed ratio for each of the six pairs of opioid combinations, which, resulted in a synergistic antinociception except for methadone/tramadol and fentanyl/tramadol which were additive, in the hot plate. The opioid antagonists naltrexone, naltrindole and nor-binaltorphimine, suggests that the synergism of morphine combinations are due to the activation of MOR subtypes with partially contribution of DOR and KOR, however fentanyl and methadone combinations are partially due to the activation of MOR and DOR subtypes and KOR lack of participation. The antinociceptive effects of tramadol combinations, are partially due to the activation of MOR, DOR and KOR opioid subtypes.

**Conclusion:**

These results suggets that effectiveness and magnitude of the interactions between opioids are dependent on pain stimulus intensity.

## Background

Opioids have been the main drugs used in the management of pain for several decades. However, repeated use of opioids, such as morphine and heroin, cause analgesic tolerance, physical dependence, and opioid addiction [1].Opioid effects are mediated by three major types of opioid receptors: μ-, δ-, and κ-opioid receptors (MORs, DORs, and KORs, respectively) [2]. Previous studies have shown that majority of opioid effects, including analgesia, tolerance, and addiction, are primarily mediated by MORs, however the effects of the other two opioid receptors remain elusive [1],[2]. Several types of analgesic combinations are available for the treatment of pain, using drugs which differ in their mechanisms of action with the aim of maximize therapeutic efficacy while minimizing side effects. Also, fixed ratio drug combinations are widely used [3]. Combinations between different non-steroidal anti-inflammatory drugs (NSAIDs) and opioids, have been evaluated either experimentally or clinically [4]-[8]. However, combinations of two opioid receptor agonists have been poorly explored experimentally [9],[10]. There is scarce information about clinical trials performed with opioid associations in patients affected by pain [11]. From the theoretical point of view, the combination opioid-opioid could induce an adequate control of pain, with a reduced doses and less adverse effects.

In this study, a tonic model of induced pain, the writhing test was selected as a model of acute visceral pain, because it seems to be a model of clinical relevancy in intestinal pain in humans [12]. The acetic acid is an easy and fast screening model to access the activity of analgesic drugs and their mechanisms. These models induce a characteristic and quantifiable overt pain-like behaviour described as a writhing response or abdominal contortion [13]. A phasic model of induced pain was also utilized, the hot plate assay [14]. In this assay, the latency to pain reflex behaviour is measured.

The purpose of this study was to evaluate the interaction between all possible combinations of the following opioids: morphine, methadone, tramadol and fentanyl, in both models of pain. The analysis of the interaction was carried out by isobolographic analysis with the calculations of the interactions indexes. Besides the role of the antagonists of different subtypes of opioid receptors in the interaction was evaluated.

## Methods

In all experiments CF-1 male mice, weighing 28–30 g, housed in a 12-h light–dark cycle at 22 ± 1°C, with free access to food and water were used. The animals were acclimatized to the laboratory environment for at least 2 h before use. Experiments were carried out in accordance with the Guide for the Care and Use of Laboratory Animals issued by the National Institute of Health, and experimental procedures were approved by the Institutional Animal Care and Use Committee at the Universidad de Chile, Santiago, Chile. Each animal was used only once, and received only one dose of the drugs tested. All drugs were freshly prepared by dissolving them in normal saline, and administered intraperitoneally (i.p.). The authors performed all observations in a randomized and blind manner. Control saline animals were run interspersed concurrently with the drug-treated animals (at least two mice per group), which prevented all the controls being run on a single group of mice at one time during the course of the research.

### Acetic acid writhing test

A modification of the method previously described was used [15]. To perform the test, mice were injected i.p. with10 mL/kg of 0.6% acetic acid solution, 30 min after the i.p. administration of the drugs, a time at which preliminary experiments showed the occurrence of the maximum effect. A writhe is characterized by a wave of contractions of the abdominal musculature, followed by the extension of the hind limbs. The number of writhes in a 5 min period was counted, starting 5 min after the administration of acetic acid. Antinociceptive activity was expressed as the maximum possible effect (% MPE) of the percent inhibition of the usual number of writhes observed in control animals ( 20.4 ± 0.36, n = 12). The dose of the drug that produced 50% of MPE (ED_50_) was calculated from the linear regression analysis of the curve obtained by plotting log dose vs percentage of MPE.

### Hot plate test

This thermal antinociceptive test was performed according with a modification to the method of Eddy and Leimbach [16]. A commercial device (Ugo Basile, Italy) was calibrated at 50 ± 0.2°C and the cut-off time was set at 30 sec. Each mouse was placed on the heated surface and the time, in sec, between placement and licking or shaking the hind paw or jumping was recorded as response latency and is a sign of thermal nociception. Each animal was tested twice before (control latency = 15.50 ± 0.40) and after the drug administration and the antinociceptive activity and was expressed as the maximum possible effect (MPE) using the formula:

(1) $$\%\mathrm{MPE}\;=\;\left[\left(\mathrm{drug}\;\mathrm{latency}\;–\;\mathrm{control}\;\mathrm{latency}\right)/\left(30\;\sec–\mathrm{control}\;\mathrm{latency}\right)\right]\;\times \;100$$

### Protocols

#### Writhing and hot plate test

Dose–response curves for i.p. administration of the following opioids: morphine, methadone, fentanyl and tramadol were obtained using eight animals with at least four doses for each. Linear regression analysis of the log dose–response curves allowed the calculation of the doses that produced 50% of antinociception (ED_50_), when each drug was administered alone. ED_50_ was used in the acetic acid writhing test or in the hot plate assay, as the equieffective dose for isobolographic analysis. Then a similar dose–response curve was also obtained and analysed after the coadministration of morphine with methadone, fentanyl and tramadol. Similarly, after the coadministration of methadone with fentanyl and tramadol, and finally, coadministration of fentanyl and tramadol. The dose–response curve was obtained by the intraperitoneal coadministration of each mixture of opioids in fixed ratio combinations of fractions of their respective ED50 values: 1/2, 1/4, 1/8, 1/16. In addition, the effect of the pretreatment on the mice with naltrexone (NTX), naltrindole (NTI) and nor-binaltorphimine (nor-BNI) in the antinociception induced by the coadministration of opioids was evaluated.

### Isobolographic analysis

Isobolographic analysis was used to characterize drug interactions. The method of isobolographic analysis has been previously described in detail [5],[17]. The isobolograms were constructed by connecting the ED_50_ of one opioid plotted on the abscissa with the ED_50_ of other opioid plotted on the ordinate, to obtain the additive line. For each mixture of opioids, the ED_50_ and its associated 95% confidence intervals (CL) were determined by linear regression analysis of the log dose–response curve (eight animals at each with at least four doses), and compared by a *t* test to a theoretical additive ED_50_ that was obtained from: ED_50_ add = ED_50_ one opioid/(P1 + R X P2 ) where R is the potency ratio of one opioid alone compared to the other opioid alone, P1 is the proportion of one of the opioids and P2 is the proportion of the other opioid in the total mixture.

In this study, fixed ratio proportions were selected, first by combining the ED_50_ of each compound, and then constructing a dose–response curve in which ED_50_ fractions (1/2, 1/4, 1/8, and 1/16) of the one opioid and the other opioid combination were administered. In the equation above, ED_50_ add is the total dose and the variance of ED_50_ add was calculated from the fraction of the ED_50_s (i.e., 0.5) in the combination as:

(2) $$\mathrm{Var}\;{\mathrm{ED}}_{50}\mathrm{add}\;=\;{\left(0.5\right)}^{2}\mathrm{Var}\;{\mathrm{ED}}_{50}\mathrm{one}\;\mathrm{opioid}\;+\;{\left(0.5\right)}^{2}\mathrm{Var}\;{\mathrm{ED}}_{50}\mathrm{the}\;\mathrm{other}\;\mathrm{opioid}$$

From these variances, confidence limits were calculated and determined according to the ratio of the individual drugs in the combination. The ED_50_ for the drug combinations was obtained by linear regression analysis of the dose–response curves. Supra-additivity or synergistic effect is defined as the effect of a drug combination that is higher and statistically different (ED_50_ significantly lower) than the theoretically calculated equieffect of a drug combination with the same proportions. If the ED_50_s are not statistically different, the effect of the combination is additive, and additivity means that each constituent contributes with its own potency to the total effect.

Furthermore, the interaction index (I.I.), or the combination potency to additive potency ratio, indicates the magnitude and nature of the interaction. I.I. It was calculated as :I.I. = experimental ED_50_/theoretical ED_50._

When the value is close to 1, the interaction is additive. Values lower than 1 are indications of the supra-additive or synergistic interactions magnitude, and values higher than 1 correspond to sub-additive or antagonisticinteractions [5],[17].

### Drugs

All drugs were freshly dissolved in saline on a constant volume of 10 ml/kg and administered as mg/kg. Morphine hydrochloride, methadone hydrochloride, fentanyl citrate, tramadol hydrochloride, naltrexone hydrochloride, naltrindole hydrochloride and nor-BNI dihydrochloride were purchased from Sigma Chemical Co, USA. Doses were expressed on the basis of the salts.

### Statistical analysis

Results are presented as a mean ± standard error of the mean (SEM) or as ED_50_ values with 95% confidence limits (95% CL). All calculations, including the statistical analysis of the isobolograms related to the difference between experimental ED_50_ and theoretical ED_50_ values by Student’s *t* test for independent means, were performed with the program Pharm Tools Pro (version 1.27; McCary Group Inc., PA, USA), and based on Tallarida [17]. *P* values under 0.05 (P < 0.05) were considered significant.

## Results

The opioid treatments in this study did not affect the functional aspect of the mice, because no abnormality was observed in the motor function after the opioid treatments, even at the highest doses used in this study.

### Antinociception induced by opioids

**Figure 1:**
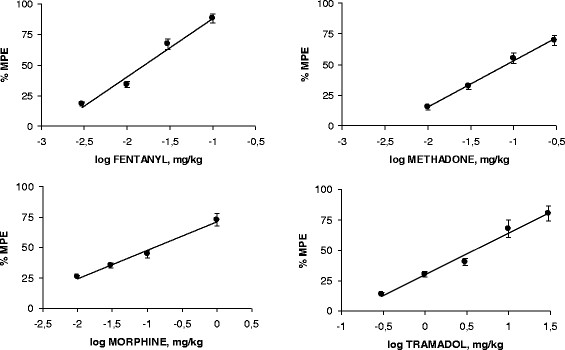
**Dose–response curves for the antinociceptive activity in the writhing test of mice induced by fentanyl, methadone, morphine and tramadol.** Each point is the mean ± SEM of six animals. % MPE = antinociception represented as a percentage of maximum possible effect.

The i.p. administration of morphine (0.01, 0.03, 0.1 and 0.3 mg/kg), displayed a dose-dependent antinociceptive activity with different efficacy in the acetic acid writhing assay of the mice, and the ED_50_ resulted to be 0.124 ± 0.018 mg/kg. The administration of methadone (0.01, 0.03, 0.1 and 0.3 mg/kg), induced a dose–response curve, and the ED_50_ was 0.005 ± 0.018 mg/kg. After the administration of fentanyl (0.001, 0.003, 0.01 and 0.03 mg/kg) the ED_50_resulted in 0.016 ± 0.002 mg/kg. The administration of tramadol (1, 3, 10 and 30 mg/kg) with an ED_50_ of 3.904 ± 0.495 mg/kg. The ED_50_ demonstrated the following rank of efficacy: fentanyl > methadone > morphine > tramadol. All these results are summarized in Figure 1 and Table 1.

**Table 1:** **ED**_**50**_**values, in mg/kg ± SEM, for the antinociceptive activity of intraperitoneal administration of fentanyl, methadone, morphine and tramadol without and with naltrexone (NTX) 1 mg/kg i.p., naltrindole (NTI) 2 mg/kg i.p. and nor-BNI 3 mg/kg i.p. in the writhing test of mice**

**Drug**	**ED**_**50 ±**_**SEM**			
	**Alone**	**+ NTX**	**+ NTI**	**+ nor-BNI**
	**(n = 24)**	**(n = 24)**	**(n = 24)**	**(n = 24)**
Fentanyl	0.016 ± 0.002	3.04 ± 0.38*	2.89 ± 0.42	0.017 ± 0.003
Methadone	0.085 ± 0.005	1.45 ± 0.15*	0.065 ± 0.008	0.081 ± 0.006
Morphine	0.125 ± 0.019	2.20 ± 0.36*	1.56 ± 0.15*	0.097 ± 0.14
Tramadol	3.904 ± 0.495	4.24 ± 0.80	2.98 ± 0.35	2.86 ± 0.65

All results between opioids alone are significant (P < 0.05). *P < 0.05 comparing with ED_50_ alone. n = number of animals used in each drug determination.

In the hot-plate test of mice, the same doses of opioids induced a dose-dependent antinociception with different efficacy. The ED_50_ values of fentanyl, methadone, morphine and tramadol were 0.040, 0.24, 1.94 and 30.53 mg/kg, respectively (see Table 2).

**Table 2:** **ED**_**50**_** values, in mg/kg ± SEM, for the antinociceptive activity of intraperitoneal administration of fentanyl, methadone, morphine and tramadol without and with naltrexone (NTX) 1 mg/kg i.p., naltrindole (NTI) 2 mg/kg i.p. and nor-BNI 3 mg/kg i.p. in the hot plate test of mice**

**Drug**	**ED**_**50 ±**_**SEM**			
	**Alone**	**+ NTX**	**+ NTI**	**+ nor-BNI**
	**(n = 24)**	**(n = 24)**	**(n = 24)**	**(n = 24)**
Fentanyl	0.040 ± 0.002	2.41 ± 0.25*	2.51 ± 0.25*	0.037 ± 0.003
Methadone	0.24 ± 0.015	0.58 ± 0.08*	0.011 ± 0.008*	0.022 ± 0.006
Morphine	1.94 ± 0.084	3.82 ± 0.36*	3.56 ± 0.15*	2.31 ± 0.44
Tramadol	30.53 ± 0.98	34.74 ± 1.25*	32.14 ± 0.53	29.75 ± 2.58

All results between opioids alone are significant (P < 0.05). *P < 0.05 when comparing con opioids alone.

The rank of potency of opioids in the writhing test to hot plate assay was from 2.5 (fentanyl) to 15.5 (morphine) times, respectively.

### Effect of naltrexone (NTX), naltrindole (NTI) and nor-BNI in the antinociception of opioids

In the writhing test, **t**he pretreatment with NTX, 1 mg/kg i.p. antagonized the antinociceptive activity of the ED_50_ of morphine, fentanyl and methadone, but partially reversed the effect of tramadol. The administration of 2 mg/kg i.p. of NTI, antagonized the antinociception induced by morphine and fentanyl, however, partially reversed the effect of methadone and tramadol. The administration of 3 mg/kg i.p. of nor-BNI, partially antagonized the antinociceptive activity of morphine and tramadol, but there was a lack of effect in the antinociception induced by fentanyl and methadone, all these results are summarized in Table 1. In the case of hot plate assay, NTX antagonized the antinociceptive activity of morphine, methadone, fentanyl and tramadol. The pretreatment with NTI antagonized the antinociceptive action of all opioids with the exception of tramadol. In addition, mice pretreated with nor-BNI lack of effect in the antinociception produced by all the opioids tested. These results are summarized in Table 2. The opioid antagonist doses were adapted or modified from previously published studies that showed the pharmacological activity of each individual receptor subtype [18]-[23].

### Interactions between opioids

The antinociceptive activity interactions of the i.p. coadministration on the basis of a fixed ratio of (1:1) combination of their ED_50_ values alone of the different opioids used in this work was evaluated by isobolographic analysis.

**Figure 2:**
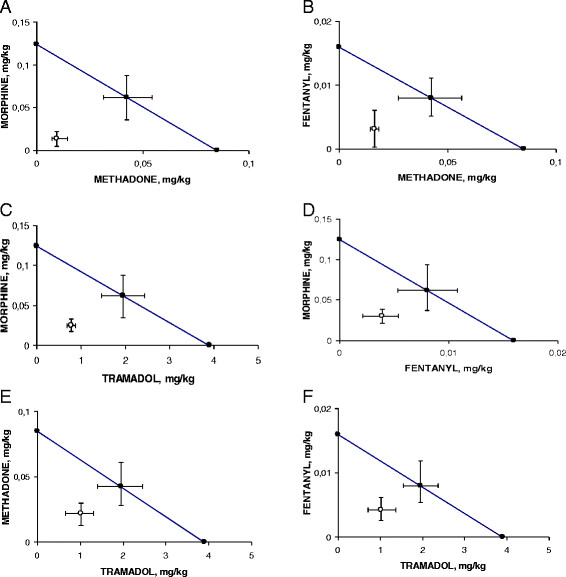
**Isobolograms for the administration of the combination of methadone with morphine (2 A), methadone with fentanyl (2 B), tramadol and morphine (2 C), fentanyl and morphine (2 D),tramadol with methadone (2 E) and tramadol with fentanyl (2 F), in the writhing test of mice.** Theoretical ED_50_ value with 95% CL (●). Experimental ED_50_ value with 95% CL (○).

The isobolograms demonstrated that all the mixtures of opioids, in the writhing test of the mice, resulted in synergistic interaction of different magnitudes as can be seen in Figure 2 (panel A-F). Table 3 shows the theoretical additive and the experimental ED_50_ values for the combinations with their 95% CL and their interaction index values.

**Table 3:** **Theoretical and experimental ED**_**50**_**values with 95% CL, and interaction index for the antinociceptive activity of the intraperitoneal coadministration of morphine with methadone, morphine with fentanyl, and morphine with tramadol, fentanyl with methadone, methadone with tramadol, and fentanyl with tramadol in the writhing test of mice**

**Drugs**	**ED**_**50**_**(95% CL)**		**Interaction index**
	**Theoretical**	**Experimental**	
Morphine/methadone	0.104(0.14-0.07)	0.023*(0.03-0.02)	0.226
Methadone/fentanyl	0.050(0.07-0.03)	0.019* (0.02-0.01)	0.387
Morphine/tramadol	2.014(3.55-1.14)	0.811* (1.05-0.64)	0.403
Morphine/fentanyl	0.070(0.10-0.05)	0.034* (0.06-0.02)	0.487
Methadone/tramadol	1.994(3.01-1.31)	1.032* (2.20-0.62)	0.518
Fentanyl/tramadol	1.960(2.88-1.32)	1.023* (1.68-0.70)	0.522

*P < 0.05 compared with ED_50_ theoretical.

Furthermore, the interaction index values of the i.p. combinations on the writhing test, demonstrated the following rank of potencies: morphine with methadone > methadone with fentanyl > morphine with tramadol > morphine with fentanyl > methadone with tramadol > fentanyl with tramadol as it is shown in Table 3.

**Figure 3:**
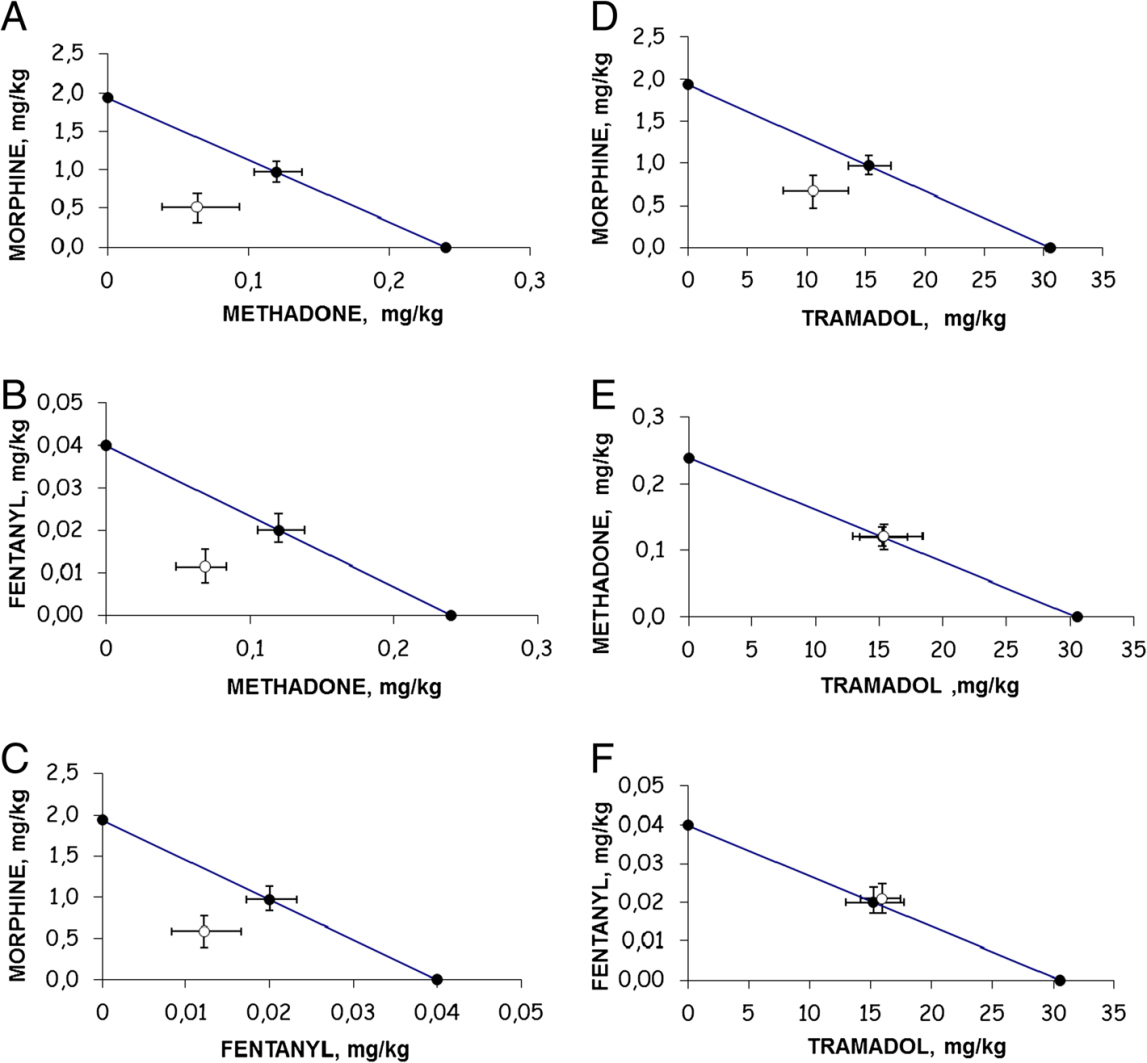
**Isobolograms for the administration of the combination of methadone with morphine (3 A), methadone with fentanyl (3 B), fentanyl and morphine (3 C), tramadol and morphine (3 D),tramadol with methadone (3 E) and tramadol with fentanyl (3 F), in the hot plate test of mice.** Theoretical ED_50_ value with 95% CL (●). Experimental ED_50_ value with 95% CL (○).

In the hot plate assay, the isobolographic analysis of the opioids coadministration on the basis of a fixed ratio of (1:1) combination of their ED_50_ values alone, resulted in a synergistic interaction between morphine with methadone, methadone with fentanyl, morphine with fentanyl and morphine with tramadol. The mixtures methadone with tramadol and fentanyl with tramadol resulted only additive, as can be seen in Figure 3 (panel A-F). In addition, Table 4 shows the theoretical additive and the experimental ED_50_ values for the different combinations with their 95% CL and their interaction index values. In this assay, the rank of potency of the interaction index was: morphine with methadone > methadone with fentanyl > morphine with fentanyl > morphine with tramadol > methadone with tramadol > fentanyl with tramadol as it is shown in Table 4.

**Table 4:** **Theoretical and experimental ED**_**50**_** values with 95% CL, and interaction index for the antinociceptive activity of the intraperitoneal coadministration of morphine with methadone, morphine with fentanyl, and morphine with tramadol, fentanyl with methadone, methadone with tramadol, and fentanyl with tramadol in the hot plate test of mice**

**Drugs**	**ED**_**50**_**(95% CL)**		**Interaction index**
	**Theoretical**	**Experimental**	
Morphine/methadone	1.09 (0.14-0.07)	0.58*(0.7-0.02)	0.532
Methadone/fentanyl	0.14(0.28-0.06)	0.08* (0.10-0.01)	0.571
Morphine/fentanyl	0.99(1.14-0.85)	0.60* (0.78-0.20)	0.606
Morphine/tramadol	16.23(18.26-14.41)	11.20* (12.71-9.64)	0.690
Methadone/tramadol	15.38(1740–13.58)	15.51 (16.85-12.62)	1.008
Fentanyl/tramadol	15.28(15.45-15.11)	16.00 (16.92-15.012)	1.047

*P < 0.05 compared with ED_50_ theoretical.

The relation between the interaction index, in the opioid mixtures changes from 1.41 in morphine with fentanyl to 2.35 in morphine with methadone, respectively, to compare both assays. In addition, the ratio of experimental ED_50_ of the opioid combinations, in the writhing to hot plate tests, varied from 4 in methadone with fentanyl to 25 in morphine with methadone, respectively.

### Effect of naltrexone (NTX), naltrindole (NTI) and nor-BNI in the interactions of opioids

Using the writhing test, NTX antagonized the antinociceptive activity of the coadministration of methadone with fentanyl, morphine with methadone, morphine with fentanyl, morphine with tramadol, methadone with tramadol, and fentanyl with tramadol. The pretreatment with NTI antagonized the antinociceptive action of the all mixture of opioids. In addition, mice pretreated with nor-BNI, the κ-opioid antagonist lack of effect in the antinociception produced by all the different combination of opioids. All these results are summarized in Table 5.

**Table 5:** **Experimental ED**_**50**_** values, without and with naltrexone (NTX) 1 mg/kg i.p., naltrindole (NTI) 2 mg/kg i.p. and nor-BNI 3 mg/kg i.p. for the antinociceptive activity of the intraperitoneal coadministration of morphine with methadone, morphine with fentanyl, and morphine with tramadol, fentanyl with methadone, methadone with tramadol, and fentanyl with tramadol in the writhing test of mice**

**Drugs**	**Experimental ED**_**50**_			
	**Alone**	**+NTX**	**+NTI**	**+ nor-BNI**
	**(n = 24)**	**(n = 24)**	**(n = 24)**	**(n = 24)**
Methadone/fentanyl	0.019	0.048*	0.21*	0.018
Morphine/Methadone	0.023	0.095*	0.089*	0.019
Morphine/fentanyl	0.034	0.061*	0.058*	0.027
Morphine/tramadol	0.811	1.05*	1.10*	0.752
Fentanyl/tramadol	1.023	1.27*	1.31*	1.010
Methadone/tramadol	1.032	1.25*	1.32*	1.024

*P < 0.05 compared with ED_50_ experimental alone.

In the hot plate assay, NTX was able to antagonize the antinociception induced by the combinations of morphine with methadone, methadone with fentanyl, morphine with fentanyl and morphine with tramadol. NTX did not induced changes in the analgesic activity of methadone with tramadol and fentanyl with tramadol. The pretreatment of the mice with NTI, reply the antagonistic activity of NTX, however nor-BNI lack of blocking action in all the combinations of opioids. See all these results in Table 6.

**Table 6:** **Experimental ED**_**50**_** values, without and with naltrexone (NTX) 1 mg/kg i.p., naltrindole (NTI) 2 mg/kg i.p. and nor-BNI 3 mg/kg i.p. for the antinociceptive activity of the intraperitoneal coadministration of morphine with methadone, morphine with fentanyl, and morphine with tramadol, fentanyl with methadone, methadone with tramadol, and fentanyl with tramadol in the hot plate test of mice**

**Drugs**	**Experimental ED**_**50**_			
	**Alone**	**+NTX**	**+NTI**	**+ nor-BNI**
	**(n = 24)**	**(n = 24)**	**(n = 24)**	**(n = 24)**
Methadone/fentanyl	0.08	0.12*	0.15*	0.07
Morphine/Methadone	0.58	0.97*	0.89*	0.49
Morphine/fentanyl	0.60	0.97*	1.02*	0.58
Morphine/tramadol	11.20	14.41*	13.45*	10.58
Methadone/tramadol	15.51	15.30	14.89	14.52
Fentanyl/tramadol	16.00	15.65	15.02	15.61

*P < 0.05 compared with ED_50_ experimental alone.

## Discussion

Opioids are the foremost drugs in the pharmacological treatment of pain.

However, they have not been compared extensively in different animal pain models. The writhing test is a model of visceral acute pain, it is very useful when drawing up directions for the therapeutic administration of drugs in humans [14]. This model was selected because it closely resembles clinical relevant acute post operatory pain in humans [24].

The results obtained in this work, demonstrate that the different opioids used in this work -- fentanyl the most potent and tramadol the least potent -- produced antinociceptive activity in the acetic acid writhing test and in the hot plate assay. The analysis of the ED_50_ values of four opioids reveal different efficacies depending on the selective opioid receptor ligands. Thus the binding potencies in nmol for MOR are 0.39 for fentanyl, 0.72 for methadone and 14 for morphine [2],[25]. In the case of the racemic tramadol, the (+) enantiomer had a binding potency value of only 1.33 and the (−) enantiomer had even lower affinity of 24.8 for MOR [26]. Also, the degree of lipophilicity could help to correlate the difference between the ED50 of the opioids. Consequently, high-efficacy ligands, such as fentanyl and methadone possesses a higher ED_50_ compared to lower efficacy ligands such as morphine with a lower ED_50_[2],[27].

The findings of this study are in agreement with previous works. So, morphine induced analgesia in acetic acid writhing test, with a dose range from 0.12 – 2.67 mg/kg [28],[29] and tramadol produced antinociceptive activity in the same test with doses between 3.9 to 14.73 mg/kg [6]. Besides, the analgesic effect produced by fentanyl was reported at 0.016 – 0.03 mg/kg, by using the same assay utilized in this work [29],[30]. In addition, methadone at 0.085 to 0.467 mg/kg, produced antinociception in the acetic acid writhing test [31]. Also, in the hot plate the findings are concordant with previous works. So, antinociceptive effects of morphine, fentanyl, tramadol and their combination it has been described [29],[32] and also the methadone induced antinociception [33].

After i.p. administration of morphine with methadone or morphine with fentanyl or morphine with tramadol; methadone with fentanyl or methadone with tramadol, and fentanyl plus tramadol in the writhing test a synergic interaction was obtained. In the hot plate assay, the same combination of opioids resulted in a synergic interaction, except of the additivity interaction induced by tramadol with methadone or fentanyl. These results corroborate that this work supports the general premises of interactions between drugs with the same effect but acting through different mechanism of action. Therefore, one possibility in these interactions could be due to different types of the MOR receptor splice variant, where each one of them may use a different downstream signalling pathway, or it may be expressed in a different anatomical region to exhibit a distinctive pharmacological profile [34]. Moreover, hetero dimerization of the MOR and other receptor may express another mechanisms in a characteristic opioid efficacy, since the intracellular signalling can be altered by receptor dimerization [35].

On the other hand, it is likely that the interactions observed when combining morphine, methadone, fentanyl and tramadol, could be related to multiple events that underlying in the mechanisms of the different efficacy among these opioids and dependent of pain stimulus.

The opioid interactions described, could occur at one or more levels of cell functions including receptors, ion channels, lipids and protein of the membrane, second messengers, protein kinases, gene induction, or others. These interaction events are dependent on the local concentration of opioids and on the nature of the nociceptive stimulus and its transduction mechanisms [36].

The administration of acetic acid solution induces nociception by increasing the levels of prostaglandins at the peritoneum [37]. The inhibition induced by opioids in the number of writhes produced by the administration of an acetic acid solution in mice in this work, suggests the involvement of peripheral pain receptors. This also help explain the interactions obtained in this study, the fact that methadone is a mixed enantiomer with not only MOR agonist but also N-methyl-D-aspartate receptor antagonist and GABA antagonists, as morphine. This condition could increase the analgesic activity of other MOR agonists like morphine, fentanyl and tramadol [38].

Additionally, the nonopioid component of tramadol, the inhibition reuptake of norepinephrine and serotonin, could play an important role in the synergism obtained in this work.

The use of opioid antagonists help to understand the interactions induced by different combinations of opiods. Thus, morphine effects are due to the activation of MOR subtypes with partially contribution of DOR and KOR subtypes. However, the combinations of fentanyl and methadone are partially due to the activation of MOR and DOR subtypes and lack of participation of KOR subtypes. The analgesic effects of the combination of tramadol with the other opiods, are partially due to the activation of MOR, DOR and KOR opioid subtypes, beside their activity on noradrenaline and serotonin reuptake.

## Conclusion

In conclusion the results from this work could be explained by the complex pharmacology of opioids, accompanied by a variation in the spectrum of opioids which could be dependent of the chemical structure, physicochemical properties, dose, receptor binding, distribution and metabolism. These results provide evidence that the effectiveness and magnitude of the interactions between opioids are dependent on the intensity of the pain stimulus in acute pain states. The use of lower doses of opioids in this work may have some utility in the clinical treatment of pain and reduce some of the adverse effects often associated with opioid administration.

## Competing interests

The authors have declared that they have no competing interest.

## Authors’ contributions

All the authors participated in planning research, in performing experiments, and were responsible for data interpretation and collaborate in writing process of the manuscript. All authors read and approved the final manuscript.
